# Author Correction: An open label randomized clinical trial of Indomethacin for mild and moderate hospitalised Covid-19 patients

**DOI:** 10.1038/s41598-022-15107-8

**Published:** 2022-06-20

**Authors:** Rajan Ravichandran, Surapaneni Krishna Mohan, Suresh Kumar Sukumaran, Devakumar Kamaraj, Sumetha Suga Daivasuga, Samson Oliver Abraham Samuel Ravi, Sivakumar Vijayaraghavalu, Ramarathnam Krishna Kumar

**Affiliations:** 1 Department of Nephrology, MIOT International, Chennai, 600089 India; 2 Adjunct Faculty, Indian Institute of Technology Madras (IITM), Chennai, Tamil Nadu 600036 India; 3 Departments of Biochemistry, Molecular Virology and Research, Panimalar Medical College Hospital and Research Institute, Chennai, Tamil Nadu 600123 India; 4 Department of General Medicine, Panimalar Medical College Hospital and Research Institute, Chennai, Tamil Nadu 600123 India; 5 Departments of Pharmacology and Research, Panimalar Medical College Hospital and Research Institute, Chennai, Tamil Nadu 600123 India; 6 Departments of Microbiology and Molecular Virology, Panimalar Medical College Hospital and Research Institute, Chennai, Tamil Nadu 600123 India; 7 Molbio Diagnostics Private Limited, Verna Industrial Estate, Verna, Goa 403 722 India; 8 School of Life Sciences, Manipur University, Imphal, Manipur 795003 India; 9 Department of Engineering Design, IITM, Chennai, Tamil Nadu 600036 India

Correction to: *Scientific Reports* 10.1038/s41598-022-10370-1, published online 19 April 2022

The original version of this Article contained an error.

Supplementary data file 1 containing anonymised patient data was inadvertently omitted.

The original Article has been corrected.

