# Supplementary material for: An open label randomized clinical trial of Indomethacin for mild and moderate hospitalised Covid-19 patients
Source: Sci Rep. 2022 Apr 19;12:6413. doi: 10.1038/s41598-022-10370-1 (PMC9016692; doi:10.1038/s41598-022-10370-1)
Supplement: Supplementary file 1 — Supplementary Information. [file 41598_2022_10370_MOESM1_ESM.pdf]

Supplementary Appendix

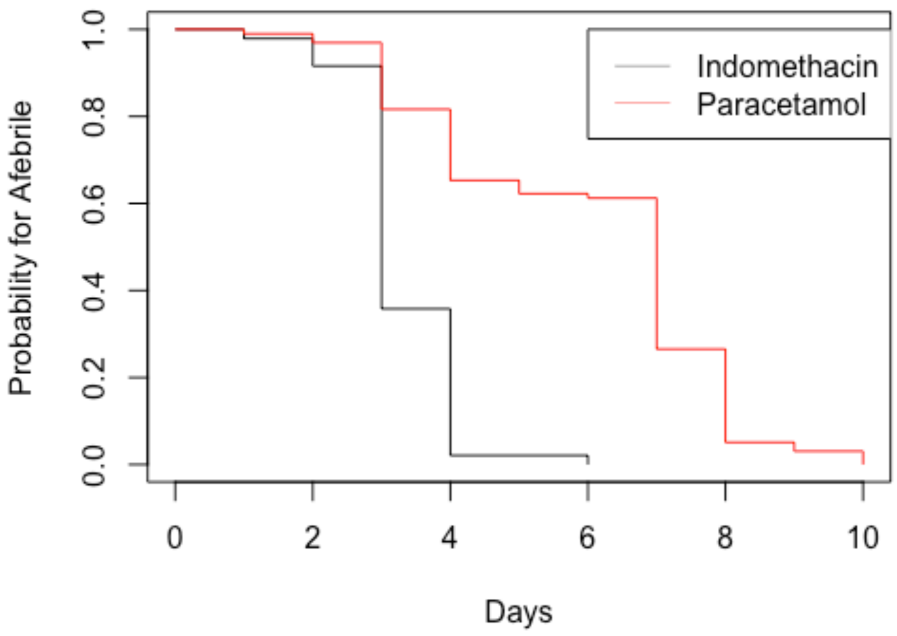

Fig. S1 Kaplan-Meir estimate for fever reduction

Covariates	Reg. Coeff	Exp (Coef)	z	Pr(>|z|)	Lower 0.95	Upper 0.95
Treatment	-2.56955	0.07657	-8.928	<2e-16	0.04356	0.1346
Age	0.013903	1.014	1.463	0.1436	0.99528	1.0331
CT Score	-0.090987	0.91303	-1.724	0.0848	0.82328	1.0126
Gender	-0.195889	0.822104	-0.878	0.3799	0.53093	1.273
CRP on admission	0.005697	1.005713	0.876	0.3812	0.99297	1.0186
Comorbidity	-0.229415	0.794999	-1.21	0.2262	0.54827	1.1528

Table S1: Cox Regression results for Fever with either indomethacin or paracetamol treatment; N=193; $N_{indomethacin}$ = 95; $N_{Paracetamol}$ = 98

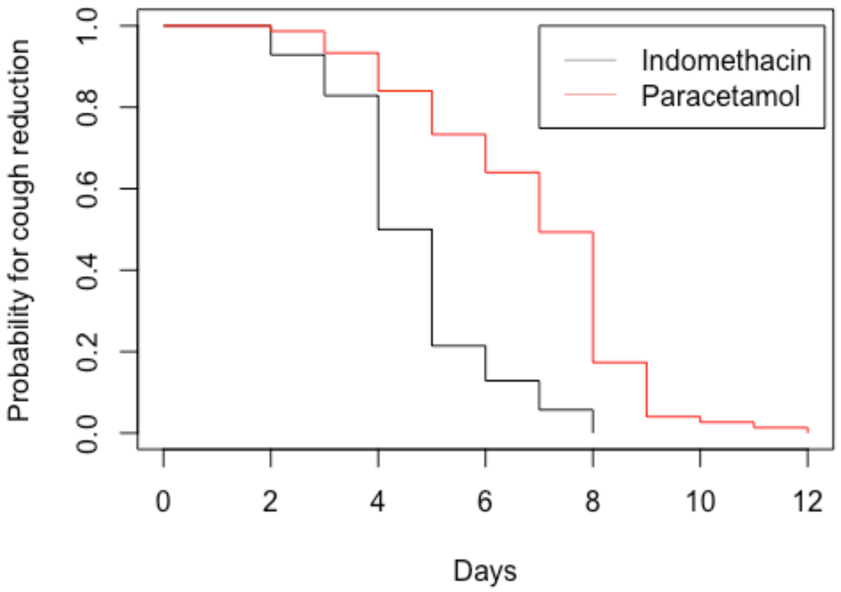

Fig S2. Kaplan-Meir estimate for cough reduction

Covariates	Reg. Coeff	exp(coef)	z	Pr(>|z|)	Lower 0.95	Upper 0.95
Treatment	-2.314587	0.098807	-7.82E+00	5.48E-15	0.0553	0.1766
age	0.012633	1.012713	1.36	0.17381	0.9944	1.0313
CT Score	-0.164531	0.848291	-2.919	0.00352	0.7596	0.9474
Gender	-0.173516	0.840704	-0.726	0.46779	0.5263	1.3429
CRP on admission	0.002738	1.002742	0.392	0.69537	0.9891	1.0166
Comorbidity	-0.030203	0.970248	-1.66E-01	0.86838	0.6788	1.3868
Cough on admission	-0.251273	0.77781	-5.104	3.32E-07	0.7063	0.8566

Table S2: Cox Regression results for Cough with either indomethacin or paracetamol treatment; N=145; $N_{indomethacin}$ = 70; $N_{Paracetamol}$ = 75

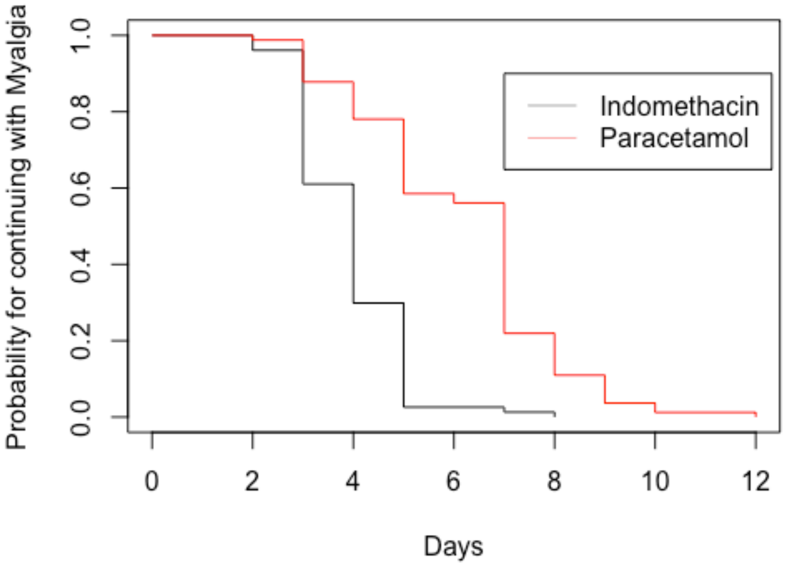

Fig S3. Kaplan – Meir estimate for continuation of Myalgia

Covariates	Reg. Coeff	exp(coef)	z	Pr(>|z|)	Lower 0.95	Upper 0.95
Treatment	-2.035077	0.13067	-7.36E+00	1.82E-13	0.07601	0.2246
Age	0.004097	1.004106	0.433	0.665	0.98565	1.0229
CT Score	-0.013054	0.987031	-0.242	0.809	0.88808	1.097
Gender	0.17193	1.187594	0.784	0.433	0.77255	1.8256
CRP on admission	0.00858	1.008617	1.258	0.208	0.99522	1.0222
Comorbidity	0.108482	1.114585	0.588	0.557	0.77626	1.6004

Table S3 : Cox Regression results for Myalgia with either indomethacin or paracetamol treatment; N=159; $N_{indomethacin}$ = 77; $N_{Paracetamol}$ = 82
